# How Genes Influence Behaviour, 2nd Edition (2020) Oxford University Press ISBN: 9,780,198,716,877 Jonathan Flint, Ralph J. Greenspan, and Kenneth S. Kendler

**DOI:** 10.1007/s10519-021-10064-w

**Published:** 2021-06-07

**Authors:** Tinca J. C. Polderman

**Affiliations:** Amsterdam UMC and Vrije Universiteit Amsterdam, Amsterdam, Netherlands

Given the extremely rapid developments of our field in the past decades, usually a textbook of behavior genetics is outdated by the time it is released. Yet, the timing of the 2nd edition of ‘How Genes Influence Behavior’ feels neat since fairly recently the behavior genetics field has made some major steps. These steps include novel developments in molecular genetic analyses and statistical applications, and, most of all, lots of exciting new results (or breakthroughs as you wish). The breadth of knowledge that is required to prepare a new generation of behavior geneticists is certainly present in the 2nd edition of ‘How Genes Influence Behavior’.

Reading ‘How Genes Influence Behavior’ often feels like sitting at the kitchen table of one of the authors who tells you various entertaining anecdotes about his long and interesting academic life in genetics. When one of the authors (KSK) describes how he collected family data in Irish families with schizophrenia in the previous century, and as such ‘came to know well (at times, perhaps too well) the bed and breakfast houses (..) of the western part of the Republic’, for example. Or, when an elderly grandmother of one of those families, wondering why KSK collects those data, clarifies that ‘everybody knows that being daft runs in families’, adding, with indignation, ‘What will these crazy Americans think of next?’. The other parts of the book are also written in a style that is informal and engaging. The historical sketch of behavior genetics, outlining the contradicting views of Mendelians versus Biometricians, is written as ‘four acts of a drama’, and indeed, it reads as a thrilling course of events. The edition further starts off typically with telling how family, twin and adoption data were used at the start of behavior genetics to study the ‘nature versus nurture’ questions and how this revealed the sources of individual differences for a variety of traits.

Subsequently, the book provides a clear section on (molecular) genetic terminology, fortunately taking into account the confusing habit in behavior genetics of using multiple terms for the same subject. What follows is an introduction into the molecular biology of genetic variation, the methods that are currently used for genotyping and sequencing, an outline of epigenetics and ‘omics’ technologies and the specific queries about inheritance that these techniques address. The authors then touch upon linkage and (candidate) genetic association analyses before fully covering genome wide association study (GWAS) developments and all ins and outs of this contemporary gene finding approach.

Having provided the readers at this point with all information needed to understand the concept of heritability, and the methodologies and results of studies in behavior genetics, the next chapters are devoted to a variety of psychological and psychiatric human traits. The authors use the different genetic architectures of these traits to address in detail the full spectrum of behavioral and molecular genetic findings, varying from family-based heritability and genetic correlations, to common and rare genetic variation, SNP heritability, and polygenic risk scores, to copy number variants, chromosomal abnormalities, mosaicism and de novo mutations. We learn that new methods, like LD regression score analysis, confirm what family studies already taught us in the early days. For example, the heritability between traits differs, and many traits are genetically correlated, sometimes in a surprising direction, like a positive genetic correlation between autism and intelligence. Another surprising finding in that context is the replicated genetic correlation of 1 between major depression and general anxiety disorder, illustrating that ‘genes often disrespect diagnostic boundaries between psychiatric disorders’.

The authors then switch gears by turning to model organisms. Studies in rodents, worms, and flies teach us a lot about reverse genetics (i.e. take the gene as starting point to investigate behavior), genetic engineering (e.g. CRISPR-Cas), and courtship (in flies). Much attention is paid to the phenotype of circadian rhythms. We get to know the ‘period gene’, and so-called clock mutants with names as ‘timeless’, ‘Clock’ and ‘shaggy’. The unusual thing about circadian rhythms is that for this phenotype large genetic effects have been detected (in contrast to most, if not all, other complex phenotypes), and the book comprehensively explains how model organism studies have contributed to this knowledge.

Since readers at this point in the book might become slightly fatigue, the authors here cleverly introduce some appealing subjects such as genes for aggression, monogamy and real estate. Again, animal studies (e.g. in voles, mice, zebra finches) are important in understanding how genes play a role in these complex behaviors. The part on animal studies ends with a section on supergenes illustrated with an anecdote of author JF, who tries to dissuade a phalanx of black ants on his kitchen floor. No need to say who owns the supergenes.

The importance of statistical and methodological rigor in contemporary genetic studies is a returning theme in almost each chapter. Apart from golden standards in measuring behavior, and applying the correct statistical tests, the problem of multiple testing is obviously addressed. Reassuring, the authors state that ‘The p-value of 5 × 10^–8^ is a value that all complex trait geneticists now have tattooed on their hearts’. Apart from statistical and methodological guidance throughout the book, Chapter 21 discusses extensively ‘how we know a finding is true’ and an excellent Appendix completes the education on genetics methodology.

In sum, the 2nd edition of ‘How Genes Influence Behavior’ is an easy to read, comprehensive, up to date textbook on the exciting field of behavior and psychiatric genetics. I am sure the entertaining writing style will enthuse many new students, as will the low price of only €43. The authors Flint, Greenspan and Kendler are to be applauded for sharing with us their fascinating journey through the world of genetic research (and Irish hostels, and tattoos), and as such delivering a great contribution to the behavior genetics literature.

